# Comparative Analysis of the Structure and Function of AAA+ Motors ClpA, ClpB, and Hsp104: Common Threads and Disparate Functions

**DOI:** 10.3389/fmolb.2017.00054

**Published:** 2017-08-03

**Authors:** Elizabeth C. Duran, Clarissa L. Weaver, Aaron L. Lucius

**Affiliations:** Department of Chemistry, University of Alabama at Birmingham Birmingham, AL, United States

**Keywords:** ClpA, ClpB, Hsp104, translocation mechanism, kinetics, thermodynamics

## Abstract

Cellular proteostasis involves not only the expression of proteins in response to environmental needs, but also the timely repair or removal of damaged or unneeded proteins. AAA+ motor proteins are critically involved in these pathways. Here, we review the structure and function of AAA+ proteins ClpA, ClpB, and Hsp104. ClpB and Hsp104 rescue damaged proteins from toxic aggregates and do not partner with any protease. ClpA functions as the regulatory component of the ATP dependent protease complex ClpAP, and also remodels inactive RepA dimers into active monomers in the absence of the protease. Because ClpA functions both with and without a proteolytic component, it is an ideal system for developing strategies that address one of the major challenges in the study of protein remodeling machines: how do we observe a reaction in which the substrate protein does not undergo covalent modification? Here, we review experimental designs developed for the examination of polypeptide translocation catalyzed by the AAA+ motors in the absence of proteolytic degradation. We propose that transient state kinetic methods are essential for the examination of elementary kinetic mechanisms of these motor proteins. Furthermore, rigorous kinetic analysis must also account for the thermodynamic properties of these complicated systems that reside in a dynamic equilibrium of oligomeric states, including the biologically active hexamer.

## Introduction

The central dogma of molecular biology tells us that proteins are constantly being produced by the cell upon exposure to environmental stresses, nutrients, and metabolites. For example, if we expose cells to a source of lactose we know that synthesis of all of the proteins responsible for lactose metabolism will be upregulated in response. However, the central dogma does not address what happens to those gene products when the lactose is gone. Indeed, the cytosol is a protein rich environment. However, every protein that was produced to respond to stimuli cannot persist in the cytosol when the stimuli are removed and the protein is no longer needed. Rebinding of repressors and removal of the mRNA are two aspects of this. Yet stemming the flow of nascent protein does not address the manner in which they are removed when new and different proteins are needed.

Generally, longer-lived proteins are sequestered into lysosomes for degradation. Shorter-lived proteins are degraded in the cytosol. The presence of a PEST region (region rich in proline, glutamate, serine, and threonine) has been associated with shorter protein half-lives (Rogers et al., 1986). The N-end rule, proposed in the 1980s and expanded upon since then, proposes that certain amino terminal residues promote ubiquitination in eukaryotes and proteolysis, two ATP-dependent processes occurring within the cytosol (Bachmair et al., 1986). Over all, cytosolic proteins can have half-lives ranging from minutes, to hours, to days (for reviews, see Dice, 1987; Varshavsky, 1996).

Proteolysis in the cytosol is a potentially dangerous activity for the cell, so removal of proteins that are no longer required presents a challenge. The cell cannot have unregulated proteolysis running rampant in the cytosol. Unregulated proteolysis in the cytosol would deplete necessary, active proteins. In fact, because dysregulation of cytosolic proteases is deadly to cells, it has been explored as an antibacterial strategy (Brotz-Oesterhelt et al., 2005; Hinzen et al., 2006).

The challenge of regulating proteolysis in the cytosol is met by ATP dependent proteases, for review see Sauer et al. (2004). However, what is the requirement for ATP in ATP dependent proteolysis? Peptide bond cleavage is exergonic. Proteases do not require an energy source to catalyze proteolysis. For example, serine proteases, cysteine proteases, aspartic proteases, etc. simply bind to a polypeptide chain and cleave the peptide bond. AAA+ (ATPases associated with a variety of cellular activities) motors and ATP serve as the regulators of proteolytic activity in the protein rich environment of the cytosol.

Across species, ATP dependent proteases are composed of a barrel shaped protease with proteolytic active sites lining the interior cavity (for review see Sauer and Baker, 2011; Olivares et al., 2016). These active sites are accessible by a pore on each end of the barrel that is too small for folded proteins to enter without first being unfolded. Certain AAA+ hexameric ring motors associate with each end of the barrel and couple the energy from ATP binding and hydrolysis to processive translocation of a polypeptide chain through the axial channel of the hexameric ring and into the proteolytic cavity of the protease. Thus, the energy source in an ATP dependent proteolytic reaction serves to both unfold the protein and processively translocate the unfolded polypeptide chain into the proteolytic chamber.

The 26S proteasome in humans and bacterial ClpAP are examples of ATP dependent proteases. ClpA is a AAA+ motor protein that contains two ATP binding sites per monomer and assembles into hexameric rings. These hexameric rings bind to one or both ends of the tetradecameric serine protease ClpP to form ClpAP. ClpA catalyzes protein unfolding and translocation of the polypeptide chain into the proteolytic cavity of ClpP.

Like proteases in the cytosol, enzyme catalyzed protein unfolding in the cytosol is potentially dangerous for the cell. However, this function emerges, or putatively emerges, in many biological contexts. For example, both ClpA and ClpX, another AAA+ motor that associates with ClpP, catalyze “protein remodeling” reactions in the absence of the proteolytic component, ClpP. ClpA remodels an inactive dimer of RepA into two active monomers (Wickner et al., 1994) and ClpX remodels the highly salt-stable MuA transposase (Levchenko et al., 1995; Kruklitis et al., 1996) to induce dissociation from DNA. More recently, mitochondrial ClpX was reported to partially unfold ALA synthase in a tranlocation-depedent mechanism to facilitate pyridoxal phosphate cofactor binding during heme biosynthesis (Kardon et al., 2015). Although it is well established that both ClpA and ClpX processively translocate a substrate into ClpP for the purposes of proteolytic degradation, it is not clear if the motors fully translocate a substrate during protein remodeling reactions. Thus, the question remains; do the motors need to fully translocate a substrate to catalyze such protein remodeling reactions? Furthermore, do they use the same elementary mechanisms to translocate substrates for proteolytic degradation as they do for protein remodeling reactions?

The AAA+ motors Katanin (McNally and Vale, 1993) and Spastin (Hazan et al., 1999) catalyze microtubule severing. Microtubule severing could also be classified as a protein remodeling reaction. It is thought that Katanin and Spastin catalyze this reaction by binding to unstructured tails on α– and β-tubulin (Roll-Mecak and McNally, 2010). Then, using the energy from ATP, they either fully or partially translocate the tubulin molecule through their axial channel. Once a monomer of tubulin is removed from the microtubule, a severing event occurs.

The N-ethylmaleimide-sensitive fusion protein (NSF) is a AAA+ motor involved in vesicle fusion (Block et al., 1988; Fleming et al., 1998; Dalal et al., 2004; Zhao et al., 2012). Specifically, the protein is responsible for disassembly of tightly associated SNARE proteins. NSF may also catalyze partial or complete unfolding/translocation in the process of dissembling the SNARE complex.

The AAA+ motors bacterial ClpB and yeast Hsp104 have the unique ability to recognize and disrupt protein aggregates *in vivo*. It has been hypothesized that these enzymes processively translocate a polypeptide chain out of a protein aggregate and through their hexameric ring structure (Weibezahn et al., 2004; Tessarz et al., 2008). However, more recent results suggest that complete translocation may not be the case (Li et al., 2015b).

One common thread among Katanin, Spastin, NSF, ClpB, and Hsp104 is that they do not interact with a protease and they are not, themselves, proteases. Thus, they do not covalently modify the substrate on which they operate. This lack of proteolytic activity leads to a technical barrier in addressing the question of whether these enzymes pass a polypeptide chain through their axial channels fully or partially. This is, in part, because unfolding alone is not evidence for complete passage. A number of studies have used GFP and its variants to examine the unfolding reaction (Weber-Ban et al., 1999; Kim Y. I. et al., 2000). However, it remains unclear how much of the GFP tertiary structure needs to be unfolded before the fluorescence is extinguished. Thus, loss of fluorescence does not allow one to conclude that complete translocation has occurred.

Complete proteolytic degradation catalyzed by ClpP is the evidence for complete translocation catalyzed by ClpA and ClpX. Much of what has been learned about translocation catalyzed by ClpA and ClpX has been determined from observing proteolytic degradation catalyzed by the protease, ClpP, in ClpAP and ClpXP, respectively. However, this leads to the question; do the motors catalyze processive translocation the same way in the absence of the proteolytic component as they do in its presence? Determining the mechanism of complete translocation catalyzed by ClpA or ClpX without covalent modification of the substrate presents the same technical difficulties as those articulated for any of the other AAA+ motors mentioned so far, i.e., the substrate on which they operate is not covalently modified.

This review is focused on efforts to examine polypeptide translocation catalyzed by AAA+ motors in the absence of proteolytic degradation. We have sought to develop a set of tools that would allow us to use transient state kinetics to examine the elementary kinetic mechanism of enzyme catalyzed protein unfolding and translocation. Specifically, we sought to determine the elementary rate constants as well as the step-size (distance per step) that define the elementary mechanism of translocation. To this end, the work began with developing strategies to examine ClpA since it was known to be a processive translocase. The work has continued by applying these approaches to the protein disaggregating machines ClpB/Hsp104. However, the work quickly revealed that in order to fully interpret the kinetic mechanistic observations a number of questions regarding the energetics of assembly and ligand binding required attention. These issues are discussed below, building on an overview of the structure of these proteins.

## Structural features of ClpA, ClpB, and Hsp104

### Primary through tertiary structure

ClpA, ClpB, and Hsp104 share similarities that have formed the basis for their classification. They are members of the AAA+ superfamily that are further classified as Hsp100 proteins for their roles in coupling ATPase activity to changes in the folding and/or assembly of substrate clients (Schirmer et al., 1996; Neuwald et al., 1999). Hsp100 members are partitioned into two classes based on the number of nucleotide binding domains (NBDs) contained per monomeric unit. Class I proteins, such as ClpA, ClpB, and Hsp104, contain two NBDs while Class II proteins, such as ClpX, contain a single NBD per monomer. In the presence of ATP, these proteins assemble into homohexameric ring-like structures that perform their chaperone activity. ATP binding and hydrolysis occur at canonical Walker A and B motifs contained within each nucleotide binding domain (Walker et al., 1982).

**Figure 1:**
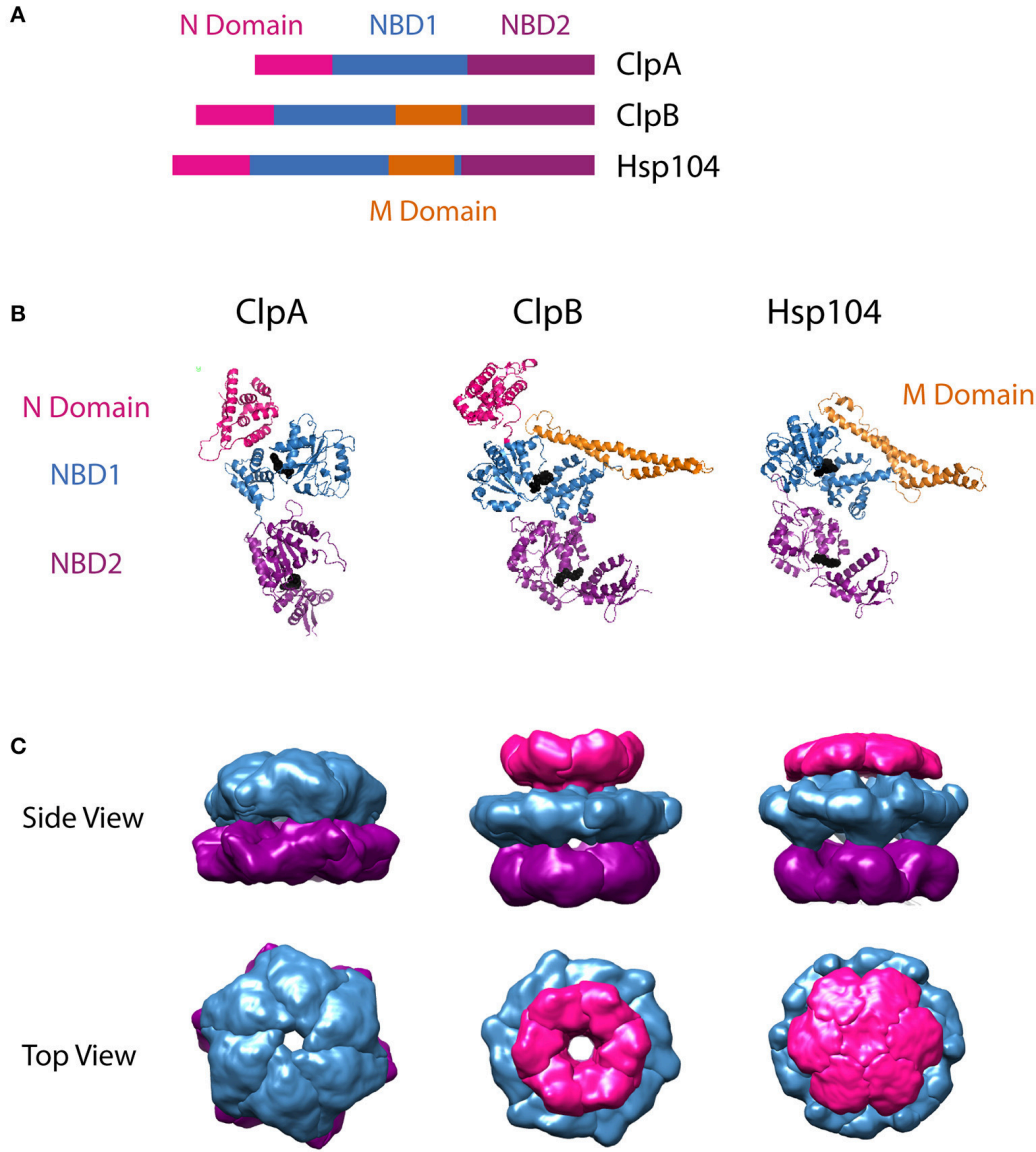
Structural comparison of ClpA, ClpB, and Hsp104. **(A)** Sequence alignment showing relative organization of N domain, NBD1, NBD2, and M domain in the AAA+ protomers compared. **(B)** Protomer crystal structures of *E. coli* ClpA (PDB ID code 1ksf) (Guo et al., 2002b), *T. thermophilus* ClpB (PDB ID code 1qvr– chain **C**) (Lee et al., 2003), and *C. thermophilum* Hsp104 (PDB ID 5d4w – chain **A**) (Heuck et al., 2016). *E. coli* ClpA and *T. thermophilus* ClpB N domains are shown in pink. *C. thermophilum* Hsp104 also has an N terminal domain, however its electron density was not resolved, likely due to flexibility. Nucleotide Binding Domain 1 (NBD1) is shown in blue for each protomer. In ClpB and Hsp104, the Middle Domain (M Domain) is shown in gold, extending in a coiled-coil from within NBD1. Nucleotide Binding Domain 2 (NBD2) is shown in purple. Bound nucleotide is shown as black spheres. These images were prepared using PyMOL Molecular Graphics System, Version 1.8 Schrödinger, LLC (Schrodinger, 2015a,b,c). Adaptation of structural comparison presented in Doyle and Wickner (2009). **(B)** Single particle reconstructions of *E. coli* ClpA (EMD-1673) (Effantin et al., 2010), *E. coli* ClpB (EMD-2563) and *S. cerevisiae* Hsp104 (EMD-2561) (Carroni et al., 2014), hexameric rings from cryo-electron microscopy. ClpA and Hsp104 models were built from images of the motor protein bound to ClpP. For Hsp104, this required use of HAP, the variant designed by the Bukau group to interact with ClpP. Top row shows views from the side. Note that the N terminal domain of ClpA was not defined in the electron density map, likely due to flexibility, similar to the observation from the crystallographic study of Hsp104. Bottom row shows views from the top, looking down through the axial channel. These images were prepared using UCSF Chimera (Computer Graphics Laboratory, University of California, San Francisco).

The protomer structures of ClpA, ClpB, and Hsp104 have been reported from various organisms in various nucleotide-bound states. In the case of ClpA, the monomer structure has been reported from *Escherichia coli* ClpA in the ADP-bound state (Guo et al., 2002b). For the disaggregases, atomic resolution crystal structures have been reported for *Thermus thermophilus* ClpB in the AMPPNP-bound state (Lee et al., 2003) and for *Chaetomium thermophilum* Hsp104 in complex with ADP (Heuck et al., 2016). Comparison of the available protomer structures (Figure 1A) as well as the primary sequences of the three motors, highlights their shared structural features. In general, each monomer is made up of an N-domain, nucleotide binding domains 1 (NBD1) and 2 (NBD2) joined by a linker region, and a C-terminal domain. The residues that separate the Walker A and Walker B motifs in each NBD have been modeled to form a loop that extends into the axial channel of the hexameric ring structures. Evidence from multiple studies has implicated conformational changes of these residues with ATP hydrolysis at each NBD in the mechanism of polypeptide substrate translocation by ClpA, ClpB/Hsp104, as discussed below in Sections Mechanisms of Polypeptide Translocation by ClpA and ClpAP and Mechanism of Translocation by ClpB/Hsp104, as well as other AAA+ motors (Yamada-Inagawa et al., 2003; Schlieker et al., 2004; Weibezahn et al., 2004; Hinnerwisch et al., 2005; Martin et al., 2008; Biter et al., 2012; Zeymer et al., 2014). As shown in Figure 1B, single particle reconstructions of the hexamer structures for the three motors predict similar arrangements of each domain within the quaternary structure. This structural similarity, in part, formed the basis for the hypothesis that ClpA, ClpB, and Hsp104 operate on substrate proteins through a shared mechanism.

ClpB and Hsp104 share an important feature that ClpA lacks. There is a middle domain (MD) located within the C-terminal end of NBD1 (Figure 1A). In the tertiary structure, this region adopts a coiled-coil fold, made up of four α-helices, that extends ~85 Å from NBD1 (Figure 1B). This domain is flexible and restriction of this flexibility has been shown to decrease disaggregation activity (Lee et al., 2003). MD flexibility has made its position and orientation within the hexamer difficult to assign in the multiple ClpB/Hsp104 structures available. The variable MD orientations in hexameric models have led to the hypothesis that nucleotide driven conformational switching of the MD may be an important part of the ClpB/Hsp104 disaggregation mechanism (Oguchi et al., 2012; Seyffer et al., 2012; Rosenzweig et al., 2013). Various studies have also shown the MD to be the binding target of ClpB/Hsp104 co-chaperones, DnaK/Hsp70 (Sielaff and Tsai, 2010; Miot et al., 2011; DeSantis et al., 2012; Seyffer et al., 2012; Lee et al., 2013; Rosenzweig et al., 2013; DeSantis et al., 2014; Doyle et al., 2015).

One other structural element distinguishes the protein translocase, ClpA, from the protein disaggregases ClpB/Hsp104: the presence or absence of a tripeptide motif requisite for the assembly with ClpP. ClpA hexamers interact with the protease ClpP through a conserved IGL/F motif nestled in a helix-loop-helix region near the C-terminal end of NBD2 (Kim et al., 2001). ClpB and Hsp104 lack that IGL/F motif, and accordingly, do not naturally associate with ClpP or any known protease.

### Quaternary structure and nucleotide-linked self-assembly

In the presence of nucleotide, ClpA, ClpB, and Hsp104 oligomerize to form homo-hexamers that interact with client substrates and partner proteins. Structural models of the hexameric state have been reported for all three motors, in various nucleotide-bound states (Guo et al., 2002b; Lee et al., 2003, 2010; Wendler et al., 2007, 2009; Effantin et al., 2010; Carroni et al., 2014; Heuck et al., 2016; Yokom et al., 2016). In most cases, the hexameric state is reported to be a planar, ring-like structure with a central axial channel as shown in Figure 1C. In these models and single-particle reconstructions, the NBDs from each protomer align side-by-side around the hexamer, forming a NBD1 tier and a NBD2 tier. Hexamer models that capture the orientation of the flexible N-domain, have a third N-domain tier above NBD1, as seen for the hexameric single particle reconstructions in Figure 1C. ClpB and Hsp104 hexamers additionally have the MD protruding from the NBD1 tier.

Recently, an alternative asymmetric spiral structure has been reported for the Hsp104 hexamer in the AMPPNP-bound state, and in the ATPγS bound state with casein bound as a substrate (Yokom et al., 2016; Gates et al., 2017) spurring interest and speculation about its structural implications for the disaggregation mechanism. Similarly, Ripstein *et al*. recently reported images of another AAA+ protein, VAT, which threads protein substrates through its axial channel into the proteasome for degradation, in transient, asymmetric conformations (Ripstein et al., 2017). These asymmetric hexameric structures observed by cryo-EM are similar to the extended spirals reported previously in crystallographic studies (Guo et al., 2002b; Lee et al., 2003; Heuck et al., 2016). These provocative asymmetric structures invite further investigation. Biochemical assays will be key in determining how the asymmetric Hsp104 spiral structure fits into the disaggregation mechanism. This and other efforts to discern the mechanistic details of substrate processing by ClpA, ClpB, and Hsp104, will require the ability to precisely quantify the concentration of hexamers competent for polypeptide substrate binding.

Many studies have established that ClpA and ClpB reside in a distribution of oligomers in the absence of nucleotide (Maurizi et al., 1998; Zolkiewski et al., 1999; Akoev et al., 2004; Veronese et al., 2009; del Castillo et al., 2011). Hydrodynamic studies from Maurizi and co-workers concluded that ClpA resides in a distribution of monomers and dimers in the absence of nucleotide and that ATP is required for assembly into hexamers (Maurizi et al., 1998). In later work, Kress et al. report that ClpA hexamerization occurs through a transient tetramer intermediate (Kress et al., 2007). Using hydrodynamic and thermodynamic techniques, it was later shown that ClpA resides in a distribution of monomers, dimers, and tetramers in the absence of nucleotide (Veronese and Lucius, 2010; Veronese et al., 2011) thereby showing that the tetramer was not a transient intermediate on the pathway to assembly but was significantly populated at thermodynamic equilibrium independent of path. Notably, in the presence of excess nucleotide, ClpA hexamers as well as lower order oligomers remain in solution (Veronese et al., 2011; Li and Lucius, 2013). However, a complete quantification of the nucleotide linked assembly reaction is still needed.

On the other hand, the energetics of ClpB self-assembly in the absence and presence of nucleotide has been quantified (Lin and Lucius, 2015b, 2016). ClpB, like ClpA, resides in a distribution of monomers, dimers, tetramers, and hexamers. An important distinction between the two motors is the observation that ClpB, unlike ClpA, forms hexamers in the absence of nucleotide. A rigorous, in-depth investigation of the self-assembly of Hsp104 is currently lacking in the field, however recent results suggest that, similar to ClpB, Hsp104 populates hexamers and lower order oligomers in both the absence and presence of nucleotide (Weaver et al., 2017). Taken together, these quantitative investigations of ClpA and ClpB self-assembly reveal that macromolecular assembly is thermodynamically linked to nucleotide binding. This has fundamental implications for the driving forces that tune the population of each oligomer in solution.

Specifically, two thermodynamic driving forces govern the self-assembly of these enzymes into hexamers: the free monomer concentration and the free nucleotide concentration. As a result, assays performed on these enzymes in which the concentrations of protein or nucleotide change throughout the experiment, must account for the changing distribution of oligomers. Failure to do so can lead to conclusions about nucleotide processing at each NBD and NBD1-NBD2 interdependence that could otherwise be explained by changes in the macromolecular state.

In much of the published work on ClpA, ClpB, and Hsp104 it has been generally assumed that in the presence of 1–2 mM nucleotide concentrations, all of the protein is in the hexameric state. This assumption is generally supported with size exclusion chromatography (SEC). However, SEC is a non-equilibrium technique, meaning that the equilibrium is perturbed by running the sample through the column. That is to say, the chemical potential of both the protein and nucleotide are changing throughout the experiment and therefore the distribution of oligomeric states is changing throughout the experiment. Moreover, the observation of hexamers in SEC does not rule out the presence of smaller oligomers. Further, it does not rule out the possibility that the self-association equilibrium has been perturbed upon introduction of a mutation in the protein.

**Figure 2:**
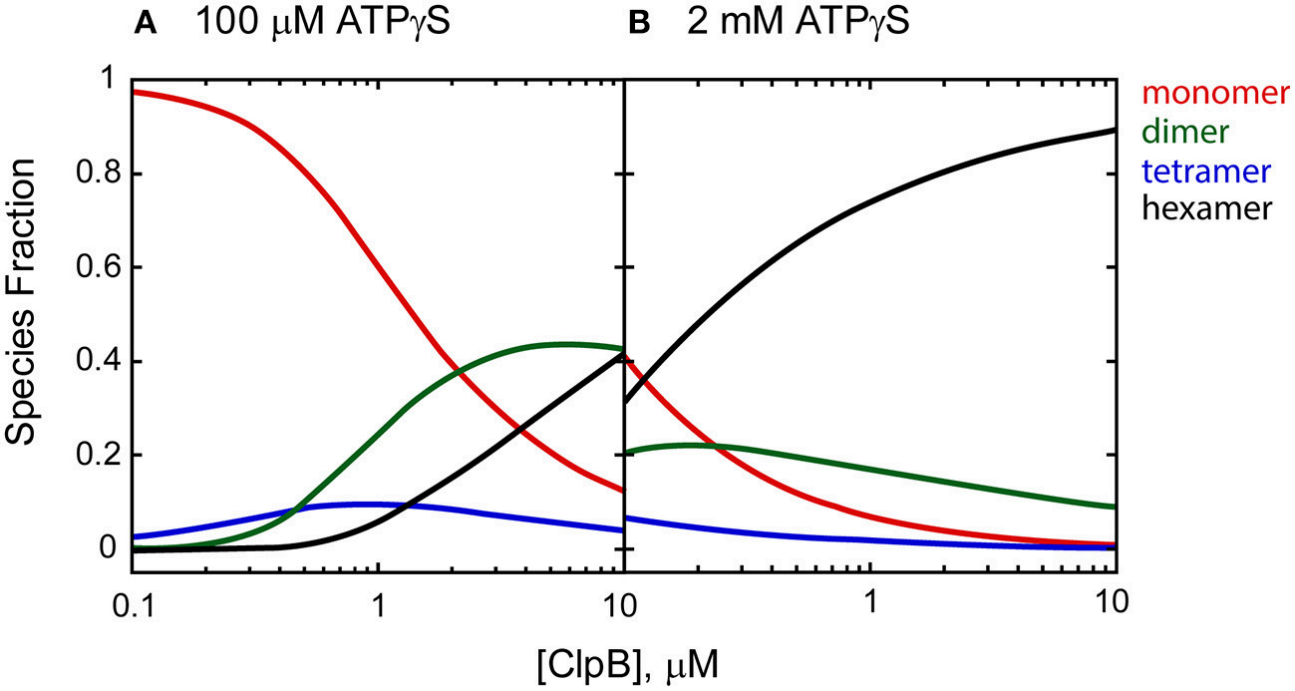
Species fraction plot as a function to total [ClpB] in monomer units. Species fractions were simulated using apparent equilibrium constants for the oligomerization of each ClpB *n*-mer (*L*_*n, app*_) predicted in the presence of **(A)** 100 μM and **(B)** 2 mM ATPγS, as well as ClpB *n*-mer nucleotide binding constants, and stoichiometries reported in Lin and Lucius (2016). The equilibrium constants for the ClpB *n*-mer oligomerization in the absence of nucleotide (*L*_*n*,0_) were used as reported in Lin and Lucius (2015b). The solid lines represent the fraction of monomer (red), dimer (green), tetramer (blue), and hexamer (black) present as a function of total [ClpB] in μM monomer.

It is clear from the self-association and polypeptide binding properties of ClpA and ClpB that smaller order oligomers do persist at saturating concentrations of nucleotide (Veronese et al., 2011; Li and Lucius, 2013; Li et al., 2015a; Lin and Lucius, 2016). For example, Figure 2 shows the fraction of ClpB oligomers populated in the presence of 100 μM and 2 mM nucleotide as a function of total [ClpB], simulated from the reported energetic parameters for ClpB assembly (Lin and Lucius, 2015b, 2016). In the presence of 100 μM nucleotide (Figure 2A), a 1 μM ClpB sample would be made up of ~6% hexamers, while 94% of the population would reside in a mixture of monomers, dimers, and tetramers. In the presence of 2 mM nucleotide (Figure 2B), the same sample would reside in a distribution made up of 74% hexamers and 26% lower order oligomers. In fact, under these conditions, even at 10 μM ClpB, the hexameric state is not fully populated, with hexamers making up ~89% of the total [ClpB]. This fact severely limits the ability to draw conclusions about the ATPase activity of the hexamer from steady state kinetic measurements where a different distribution of oligomers is present at each substrate concentration.

The simplest explanation for why the assumption that all motor protein is in the hexameric state is problematic, is that the Michaelis-Menten equation is scaled linearly by the total enzyme concentration, i.e., *V*_*max*_ = *k*_*ca*__*t*_ × *E*_0_. Recall, *E*_0_ is the total amount of enzyme in the experiment, which is controlled by the experimentalist, whereas, *E* is the free (unbound) enzyme concentration at any given time and its concentration is unknown by the experimentalist. Thus, the maximum velocity is measured at saturating substrate concentration and divided by the known total enzyme concentration, *E*_0_, and the *k*_*cat*_ is reported.

It is important to recall that *V*_*max*_ = *k*_*cat*_ × *E*_0_ emerges from two assumptions in the derivation of the Michaelis-Menten equation. The first is that the substrate is in large excess over the enzyme. The total substrate concentration relative to the total enzyme concentration is controlled by the experimentalist but, mathematically, it results in being able to assume [*S*]*k*_1_ is a constant in the first differential equation given by Equation (1) for Scheme 1.

(1) $$\begin{array}{l} \frac{d\left[ES\right]}{dt}=\left[E\right]\left[S\right]k_{1}-\left(k_{2}+k_{3}\right)\left[ES\right]\\ \;E+S\overset{k_{1}}{\underset{k_{2}}{⇌}}ES\overset{k_{3}}{\to }E+P \end{array}$$

#### Scheme 1

The second assumption, which is based on the first is that because the substrate is in large excess of the enzyme, the concentration of *ES* is considered constant or “in the steady-state.” If *ES* is constant, then the differential equation above is set to zero and solved algebraically for *ES*. However to do this the free enzyme term must be replaced with *E*_0_ − *ES*. This assumption is valid if and only if the [*ES*] is constant, which is our underpinning assumption. Thus, under constant *ES* conditions the conservation of mass equation can be rearranged to *E* = *E*_0_ − *ES*.

The assumption that the total enzyme, *E*_0_, is equal to the free enzyme, *E*, plus the bound enzyme, *ES*, only holds for a non-dissociating macromolecule. Understandably, this was not pointed out by Michaelis and Menten. However, we have not seen it expressly stated since.

The assumptions hold for self-associating systems that do not reside in dynamic equilibria, for example, if *E* forms only dimers and does not dissociate into monomers. Alternatively, if the experimentalist can maintain the concentration of the enzyme in large excess over the dimerization dissociation equilibrium constant then it may be possible to assume only dimers reside in solution. However, one has to be certain that by doing this they do not simultaneously violate the assumption that the substrate is maintained in large excess over the enzyme concentration.

If the dimer exists in a dynamic equilibrium between monomers and dimers, and concentrations of enzyme below the dimerization equilibrium constant are used, then the assumption is violated. The issue is made much more complicated for ClpA and ClpB where we, and others, have shown that both enzymes reside in a mixture of monomers, dimers, tetramers, and hexamers (del Castillo et al., 2011; Veronese et al., 2011; Lin and Lucius, 2015b, 2016). Moreover, the populations of these species are governed by the free concentration of the substrate (nucleotide). Consequently, the Michaelis-Menten equation will not be scaled by a simple relationship like *k*_*cat*_ × *E*_0_. This is because the simplest relationship that one can write down that relates the known total monomer concentration to the species that reside in solution for a system such as ClpA or ClpB is given by Equation (2):

(2) $$\begin{array}{l} E_{0}=E+2E_{2}+4E_{4}+6E_{6}+\sum_{i=1}^{2}ES_{i}+2\sum_{i=1}^{4}E_{2}S_{i}\\ \;+\;4\sum_{i=1}^{8}E_{4}S_{i}+6\sum_{i=1}^{12}E_{6}S_{i} \end{array}$$

where the subscript on *E* represents the oligomeric state and the subscript on *S* represents the number of nucleotides bound to that oligomer, represented with the counting index, *i*. There is no simple algebraic way to express Equation (2) to replace *E* in the differential equation given by Equation (1). Indeed, if no other oligomers are in solution then Equation (2) simplifies to Equation (3):

(3) $$E_{0}=6E_{6}+6\sum_{i=1}^{12}E_{6}S_{i}$$

Equation (4) is typically applied to the analysis of steady-state ATPase experiments on ClpA and ClpB. The total monomer concentration is divided by six, the *V*_*max*_ is measured and divided by *E*_0_/6 and a *k*_*cat*_ is reported.

(4) $$\frac{E_{0}}{6}=E_{6}+\sum_{i=1}^{12}E_{6}S_{i}$$

But what does this parameter mean when we know that the system resides in a dynamic equilibrium and the total enzyme concentration is actually given by Equation (2)? The answer may be that the *k*_*cat*_ is not that meaningful because it has been acquired by dividing *V*_*max*_ by a concentration that does not reflect the true hexamer concentration. However, the *V*_*max*_ itself contains meaningful information. Contained within the *V*_*max*_ is information about the self-association equilibrium constants and the nucleotide binding constants. This is because the concentration terms in Equation (2) can be replaced with the appropriate self-association equilibrium constants and the nucleotide binding constants given by Equation(5):

(5) $$E_{0}=\left[E\right]P_{1}+2L_{2,0}{\left[E\right]}^{2}P_{2}+4L_{4,0}{\left[E\right]}^{4}P_{4}+6L_{6,0}{\left[E\right]}^{6}P_{6}$$

where *L*_2,0_, *L*_4,0_, and *L*_6,0_ represent the self-association equilibrium constants for the formation of dimers, tetramers, and hexamers in the absence of nucleotide, respectively. The first subscript represents the oligomeric state and the second subscript represents the number of nucleotide bound, *E* and *E*_0_ are as above, and *P*_1_, *P*_2_, *P*_4_, and *P*_6_ are the partition functions for nucleotide binding to the monomer, dimer, tetramer, and hexamer, respectively. Each of the partition functions are functions of the nucleotide binding equilibrium constants and the free nucleotide concentration. Although there are many forms that the partition functions could take, one example for binding to the monomer could be given by Equation (6):

(6) $$P_{1}=\left(1+K_{1}\left[ATP\right]+K_{1}K_{2}{\left[ATP\right]}^{2}\right)$$

where *K*_1_ and *K*_2_ would represent the equilibrium constants for binding to NBD 1 and 2, respectively. This leads to the conclusion that if one observes differences in the *V*_*max*_ for various point mutations in the enzyme, especially mutations in the ATPase active site, then there are three potential explanations. The first is that the activity has been affected, which is the typical interpretation. However, the second and third explanation are that the nucleotide binding affinity or the self-association equilibrium has been affected by the mutation. If the mutation has perturbed the self-association equilibrium and/or the nucleotide binding affinity, then a series of comparisons on ATPase activity between variants and wild type enzymes at the same fixed protein concentration are not reporting on the same concentrations of hexamers catalyzing ATP turnover. Again, showing that hexamers still form upon introduction of mutation does not show that the self-association reaction has not been perturbed.

The resolution to this problem is to employ a thermodynamically rigorous technique that would allow one to measure the equilibrium constants and accurately predict the concentration of the active species in solution (Lin and Lucius, 2015a,b; Lin and Lucius, 2016). In other words, define the thermodynamic parameters in Equation (5) and use them to interpret the kinetic/mechanistic data. In general, the apparent self-association constant for the ligand linked assembly of ClpB would be given by Equation (7):

(7) $$L_{6,app}=\frac{\left[ClpB_{6}\right]+\sum_{i=1}^{12}\left[ClpB_{6}ATP\gamma S_{i}\right]}{{\left(\left[ClpB_{1}\right]+\sum_{i=1}^{2}\left[ClpB_{1}ATP\gamma S_{i}\right]\right)}^{6}}=\frac{\left\{ClpB_{6}\right\}}{{\left\{ClpB_{1}\right\}}^{6}}$$

where the numerator represents the summation of all of the nucleotide ligation states of hexameric ClpB in solution and the denominator represents all of the nucleotide ligation states in the monomeric state. The curly braces on the right hand side of Equation (7) are used as a shorthand notation for the summation on the left. Equation (7) can be simplified to Equation (8):

(8) $$L_{6,app}=L_{6,0}\cdot \frac{P_{6}}{{\left(P_{1}\right)}^{6}}$$

where *L*_6,0_ is as above, the hexamerization equilibrium constant in the absence of nucleotide, and *P*_6_ and *P*_1_ are the partition functions for nucleotide binding to the hexamer and the monomer, respectively. We showed, for ClpB (Lin and Lucius, 2016), that the apparent hexamerization equilibrium constant is given by Equation (9):

(9) $$L_{6,app}=L_{6,0}\cdot \frac{{\left(1+\kappa_{6}\cdot {\left[ATP\gamma S\right]}_{f}\right)}^{m_{6}}}{{\left({\left(1+\kappa_{1}\cdot {\left[ATP\gamma S\right]}_{f}\right)}^{m_{1}}\right)}^{6}}$$

where the partition functions for nucleotide binding to the hexamer and the monomer in Equation (8) are given by the partition functions for the n-independent and identical sites model, a model that is commonly used to analyze ITC data and was applied to ITC data for ClpB binding ADP (Carroni et al., 2014). In this model *k*_1_and *k*_6_ are the average step-wise equilibrium constants for nucleotide binding, *m*_1_ and *m*_6_ are the stoichiometries of binding to monomers and hexamers, respectively. In a thermodynamically rigorous and model independent analysis of our data we showed that 12 ATPγS molecules were bound to hexameric ClpB and one ATPγS was bound to the monomer. *L*_6,0_ was determined in an analysis of assembly in the absence of nucleotide (Lin and Lucius, 2015b) and from an analysis of the dependence of *L*_6,*app*_ on ATPγS we determined κ_6_ and κ_1_ (Lin and Lucius, 2016).

What is most striking, telling, and predictive about Equations (8) and (9) is that they are the simple product of two terms, the hexamerization equilibrium constant in the absence of nucleotide multiplied by the ratio of partition functions for nucleotide binding. If one seeks to introduce a mutation into a protein like ClpB then these are the parameters to interrogate. The mutation would have the ability to influence *L*_6,0_, which represents the intrinsic propensity of the protein to assemble into hexamers. However, more likely, introduction of a mutation, especially one in the ATPase active site is likely going to influence the affinity for nucleotide. It seems highly unlikely that the affinity for nucleotide binding to the hexamer, *k*_6_, would not change upon introduction of a mutation in the ATP binding site. Whether the intrinsic propensity of the enzyme to assemble or the nucleotide binding affinity is perturbed Equation (9) predicts that the concentration of hexamers in solution will be affected.

The unanswered question we now seek to address is how do partner proteins influence this equilibrium? A hallmark of AAA+ protein unfoldases is that they interact with partner proteins. ClpA interacts with the protease, ClpP and various adaptor proteins. ClpB interacts and collaborates with the KJE system and Hsp104 collaborates with Hsp70 and Hsp40. Equation (9) predicts that if these protein-protein interactions perturb the nucleotide binding by either modulating the stoichiometry or affinity then this will perturb the hexamerization equilibrium constant and thereby the concentration of hexamers present in solution. It is tempting to assert that partner proteins like ClpP and the KJE system would stabilize the hexamers. However, for a ligand linked assembling system, Equation (9) informs us that the interaction could stabilize or destabilize. In fact, since the nucleotide concentration in the cell is well above the affinity constant, here we hypothesize that the ability of partner proteins to modulate the nucleotide binding affinity allows for fine control over the concentration of hexamers present and available to do work. With a detailed analysis of ClpB assembly, we now stand poised to determine how the KJE system influences self-association.

Similarly, several groups have reported that the steady-state ATP hydrolysis rate for ClpA is reduced in the presence of ClpP (Kress et al., 2009; Baytshtok et al., 2015). In addition to ClpP exerting allosteric control over the rate of ATP hydrolysis, again, Equation (9) predicts that this phenomenological observation could be due to many factors. Our transient state kinetics experiments have suggested that ClpA uses only the NBD2 ATPase sites to catalyze processive translocation when associated with ClpP (Miller et al., 2013; Miller and Lucius, 2014). This observation does not rule out the possibility that NBD1 is still binding to ATP. However, when combined with the predictions from Equation (9) it does suggest that if the system goes from a stoichiometry of binding of 12 to 6 then this would perturb the hexamer concentration. Thus, the reduction in the steady-state ATPase rate could be due to a two-fold reduction in the binding stoichiometry and thereby a reduction in the concentration of free hexamers. Alternatively, if ClpP does stabilize the hexameric form then one would have to conclude that the elevated rate of ATP hydrolysis observed in the absence of ClpP must be due to a significant population of monomers, dimers, and tetramers rapidly hydrolyzing ATP.

The coordination of NBD1 and NBD2 has been, and continues to be, an area of great interest in the field. The use of these and other similar variants, abolishing ATP binding (Walker A) or hydrolysis (Walker B or Sensor 1) have been used by many groups to investigate the coordination of the 12 ATP binding and hydrolysis sites within a the ClpA hexamer, as well as for the ClpB and Hsp104 hexamer. One common strategy is “mutant doping,” in which a variant is added to wild type protein in known ratios (Werbeck et al., 2008; Hoskins et al., 2009; del Castillo et al., 2010; DeSantis et al., 2012; Yamasaki et al., 2015). Many conclusions have been drawn regarding sequential, probabilistic, or concerted ATP hydrolysis mechanisms. Although the statistical distribution of the number of mutant protomers contained within a hexamer is valid, it may not hold if the mutation perturbs the assembly equilibrium. Many of these studies suffer from the assumption that the entire population of protein resides in the hexameric state. The most convincing among them are experiments where the signal is only sensitive to the hexameric form. For example, it seems clear that ClpX invokes a stochastic model since the studies used a linked hexamer (Martin et al., 2005; Cordova et al., 2014). Consequently, the issues surrounding assembly have been removed.

## Mechanisms of polypeptide translocation by ClpA and ClpAP

### ClpA mechanism in the absence of ClpP

Horwich and coworkers showed that ClpAP could catalyze global unfolding of an SsrA tagged GFP construct (Weber-Ban et al., 1999). This was done by incorporating the 11 amino acid SsrA tag, which is a known binding sequence for ClpA and ClpX, at the carboxy-terminus of GFP (Levchenko et al., 1997). When the GFP-SsrA construct was presented to ClpA in the presence of ATP, a slight decrease in fluorescence was observed. However, when the construct was presented to ClpAP in the presence of ATP, a near complete loss of fluorescence was observed. This was interpreted to mean that when ClpA unfolded the GFP in the absence of a protease, GFP was allowed to spontaneously refold. However, in the presence of the proteolytic component, GFP was degraded and thus complete loss of fluorescence was observed.

To examine directional translocation catalyzed by ClpA, Horwich and coworkers developed a FRET based assay (Reid et al., 2001). In this design, a donor fluorophore was placed in the central cavity of ClpP and an acceptor at various positions on model substrates all containing the SsrA sequence at the carboxy-terminus. If ClpA translocates the polypetide chain into the ClpP cavity from the SsrA sequence at the carboxy-terminus directionally to the amino-terminus, then FRET time courses would reveal this. FRET time courses were consistent with processive translocation from the carboxy-terminus to the amino-terminus. The results clearly showed that ClpA drives translocation of a polypeptide chain into the proteolytic chamber of ClpP.

Until recently, the elementary kinetic parameters governing this translocation reaction had not been reported. Moreover, most of the mechanistic investigations available were performed in the presence of ClpP. Thus, the critically important elementary kinetic mechanism for polypeptide translocation catalyzed by ClpA was missing from the field. Determining this mechanism required the development of techniques that would be sensitive to the elementary steps in polypeptide translocation in the absence of proteolytic degradation. Such approaches could then be broadly applied to a variety of enzymes that do not associate with proteases (see examples in the Introduction). This kinetic mechanism would include the elementary rate constants governing the reaction, kinetic step-size (amino-acids translocated between two rate-limiting steps), processivity (probability the enzyme will translocate vs. dissociate), and directionality (C to N vs. N to C).

**Figure 3:**
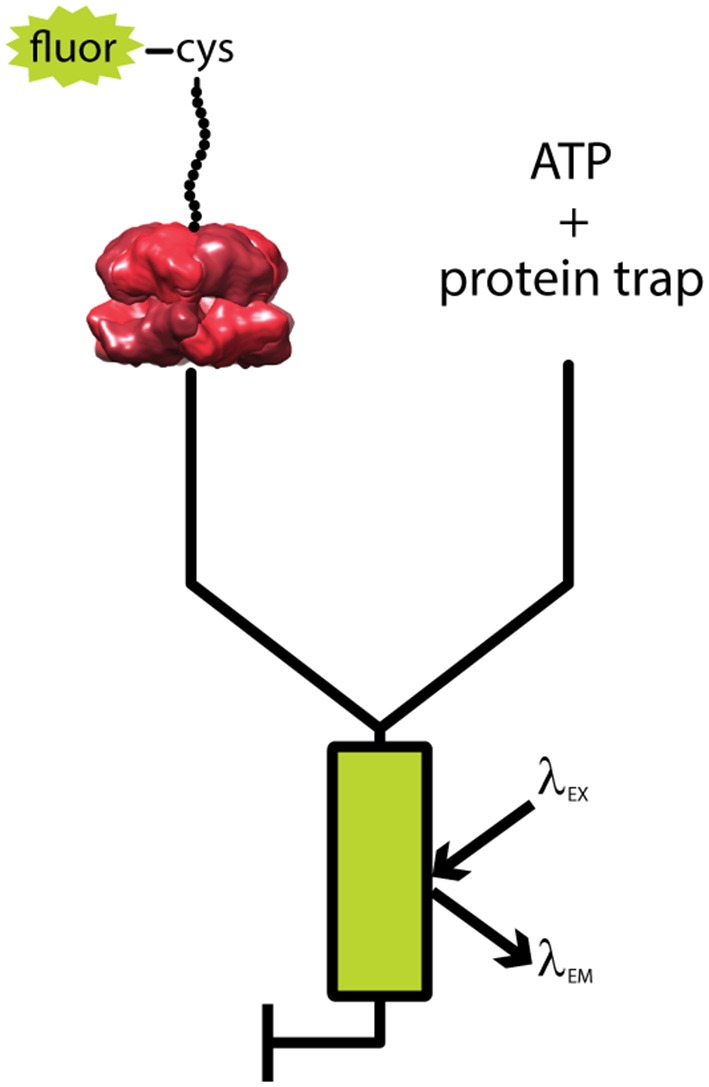
Schematic of single turnover fluorescence stopped-flow experiment. ATPγS-bound ClpA is pre-assembled with a fluorescently labeled, unstructured polypeptide substrate, fluor-peptide. The fluor-peptide bound ClpA complex (left) is then rapidly mixed with a solution of ATP and a non-fluorescent peptide (protein trap, right) held in large excess over the fluorescently modified peptide concentration. Upon mixing, any ClpA hexamers that dissociate from the fluorescently modified peptide will be swiftly bound by protein trap, ensuring the reaction monitored is single-turnover with respect to the fluor-peptide bound ClpA complex. The mixture is excited at a specified fluorophore excitation wavelength (λ_EX_), and fluorescence emission at an indicated fluorophore emission wavelength (λ_EM_) is monitored as a function of time.

A single-turnover fluorescent stopped flow assay was developed to elucidate these kinetic parameters (Rajendar and Lucius, 2010; Lucius et al., 2011). Figure 3 shows a generalized schematic representation of this rapid mixing assay. Synthetic polypeptide substrates containing the 11 amino acid SsrA binding sequence at the carboxy-terminus and a single cysteine at the amino-terminus were constructed. The sequence of the polypeptide was based on the Titin I27 domain because the long term goal was to move to full length tandem repeats of I27 as had been done for ClpX (Kenniston et al., 2003, 2005). The cysteine was labeled with fluorescein-5-maleimide. ClpA was bound to the SsrA sequence in the presence of the slowly hydrolysable ATP analog, ATPγS. Upon ClpA binding, fluorescence quenching was observed. Fluorescence quenching has since been observed for binding by both ClpB and Hsp104 to their respective substrates (Li et al., 2015b; Weaver et al., 2017). This sample was then loaded into one syringe of the stopped-flow fluorometer (see Figure 3). In the other syringe was loaded a large excess of ATP and unlabeled SsrA peptide to serve as a trap for ClpA, i.e., any free ClpA would rapidly bind to SsrA and not the fluorescently modified polypeptide (Rajendar and Lucius, 2010). The large excess of trap ensures single-turnover conditions with respect to the complex of ClpA bound to fluorescently labeled peptide.

In the single-turnover fluorescence assay, the two solutions are rapidly mixed within 2 ms in a stopped-flow fluorometer and fluorescence is observed as a function of time. Fluorescence was observed to increase with time indicating that ClpA dissociated from the polypeptide chain. The question is; do the kinetic time courses yield information on translocation before ClpA dissociates? In principle, if ClpA is taking multiple steps before dissociating then the observed kinetic time courses should reflect the number of steps the enzyme takes before dissociation. Thus, if the length of the peptide is increased, the number of steps the enzyme takes before reaching the end should also increase. That is to say, if the time courses are sensitive to processive translocation, then the time courses should depend upon substrate length.

To test the substrate length dependence of the kinetic time courses, time courses were collected as a function of polypeptide substrate length ranging from 30 to 50 amino acids (Rajendar and Lucius, 2010). Observed was a lag (constant fluorescence) followed by an increase in fluorescence. This lag was observed to increase in duration with increasing substrate length indicating that ClpA remained on the polypeptide for an increasing amount of time with increasing substrate length. This observation is interpreted to indicate that ClpA is taking more steps with each increase in substrate length. Therefore, the single-turnover fluorescence stopped-flow assay is sensitive to processive translocation.

To elucidate the elementary rate constants using transient state kinetics one needs to perturb the system. Variables like temperature, salt concentration and type, pH, etc. can be used for this perturbation. For a molecular motor that couples ATP binding and hydrolysis to repeated rounds of translocation, the simplest perturbation is to vary the ATP concentration. The initial experiments are usually carried out at excess ATP so that it can be assumed that ATP binding is not rate-limiting. As the [ATP] is reduced, the observed rate constant will reflect ATP binding, or a step coupled to ATP binding. Importantly, because the motor-peptide complex is preassembled prior to rapidly mixing with ATP (Figure 3), the signal is insensitive to the changing population of ClpA hexamers throughout the ATP range assayed. The kinetic time courses were collected as a function of ATP from ~125 μM to 5 mM. As the [ATP] was reduced, the observed kinetic rate constant decreased. This is further evidence that the time courses are reporting on translocation since simple dissociation would not be predicted to be ATP concentration dependent.

The kinetic time courses were subjected to global non-linear-least-squares (NLLS) analysis (Lucius et al., 2003, 2011). For ClpA, the enzyme translocated with a repeating rate constant, *k*_*t*_ = (1.39 ± 0.06) s^−1^ and an overall rate of (19 ± 1) AA s^−1^ at saturating ATP with a kinetic step-size of (14 ± 2) AA step^−1^. It is important to note that the kinetic step-size represents the average number of amino acids translocated between two rate-limiting steps and may or may not represent physical stepping. While similar strategies have been successfully used to examine helicase catalyzed DNA unwinding and single strand DNA translocation (Fischer and Lohman, 2004; Fischer et al., 2004; Lucius et al., 2004; Lucius and Lohman, 2004), this was the first step-size reported for a polypeptide translocase (Rajendar and Lucius, 2010; Lucius et al., 2011).

**Figure 4:**
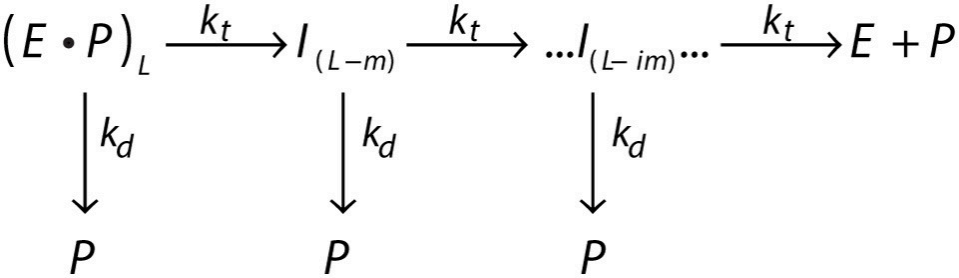
General scheme of a translocating enzyme mechanism. Translocating enzyme (*E*) in complex with a peptide (*P*) of length *L*, (E·P)_*L*_, will either translocate the peptide through a translocation rate constant (*k*_*t*_) to form an peptide intermediate translocated by a some distance *m, I*_(*L*−*m*)_, or dissociate from the peptide through a dissociation rate constant (*k*_*d*_). The translocase proceeds through multiple translocation steps of a given step-size (*m*) until the peptide is fully translocated.

The processivity is quantitatively defined as the rate constant for translocation divided by the summation of the rate constants for translocation and dissociation. For example, a translocating enzyme following the mechanism shown in Figure 4, where *E*·*P* represents the enzyme pre-bound to a peptide of length *L*, the enzyme can proceed forward with rate constant *k*_*t*_ or dissociate with rate constant *k*_*d*_. *I*_(*L*−*m*)_ represents the first intermediate that has been translocated by some distance *m* (step-size).

The processivity is the probability given by Equation (10) (Lucius et al., 2003, 2011).

(10) $$P=\frac{k_{t}}{k_{t}+k_{d}}$$

When *k*_*d*_ = 0, then *P* = 1 and every enzyme that binds will translocate to the end without dissociation. On the other hand, as *k*_*d*_ increases, *P* approaches zero, which would describe an enzyme with low processivity (an enzyme that has a higher propensity to dissociate than reach the end of the polypeptide chain). The processivity described as a probability, *P*, can be related to processivity expressed in terms of the average number of amino acids translocated per binding event, *N*, given by Equation (11) (for a complete derivation of Equation (11) see Appendix B of Lucius et al., 2003).

(11) $$P=e^{-\left(m/N\;\right)}$$

It is tempting to assume that a hexameric ring motor that encircles the linear lattice on which it translocates would be highly processive. However, this is not always true. For example, the hexameric ring helicase, DnaB exhibits a processivity of P ~0.89 (Galletto et al., 2004). The proposed model is that the ring opens and substrate can “escape” thereby resulting in a dissociation event. However, this primary replicative helicase likely exhibits much higher processivity in the context of the fast moving replication fork, likely due to interactions with other proteins. With respect to ClpB and Hsp104, both enzymes have been proposed to be in “rapid subunit exchange” (Werbeck et al., 2008; DeSantis et al., 2012). Thus, loss of a subunit in a hexameric ring could also result in a dissociation event. Moreover, like DnaB, partner proteins are likely to influence the processivity. Regardless of the mechanism, there is a dearth of quantitative measurements of processivity for polypeptide translocases.

In the initial examination of ClpA catalyzed polypeptide translocation with synthetic peptides, a measureable dissociation rate constant, *k*_*d*_, was not detected above 500 μM ATP. However, at 300 μM ATP and below, a measureable dissociation rate constant was observed, allowing for the calculation of processivity. The processivity was determined to be *P* = (0.876 ± 0.006) at low [ATP]. Using Equation (11) a processivity of ~100 amino acids per binding event is predicted, which is 2-fold larger than the longest polypeptide used in this study. Thus, this is a preliminary estimate of the processivity at limiting [ATP] and methods allowing the examination of longer polypeptides are needed to rigorously test the processivity for this and related enzymes. Qualitatively, the findings support the idea that ClpA is highly processive, confirming that reported by Maurizi and coworkers (Thompson et al., 1994).

### Effect of ClpP on the translocation mechanism catalyzed by ClpA

With a method in hand that is sensitive to polypeptide translocation in the absence of proteolytic degradation the question that could be addressed is, does ClpAP translocate using the same mechanism as ClpA alone? A qualitative assessment of stopped-flow time courses had been reported previously that concluded ClpAP translocated faster than ClpA alone but rate constants were not reported (Kolygo et al., 2009).

**Figure 5:**
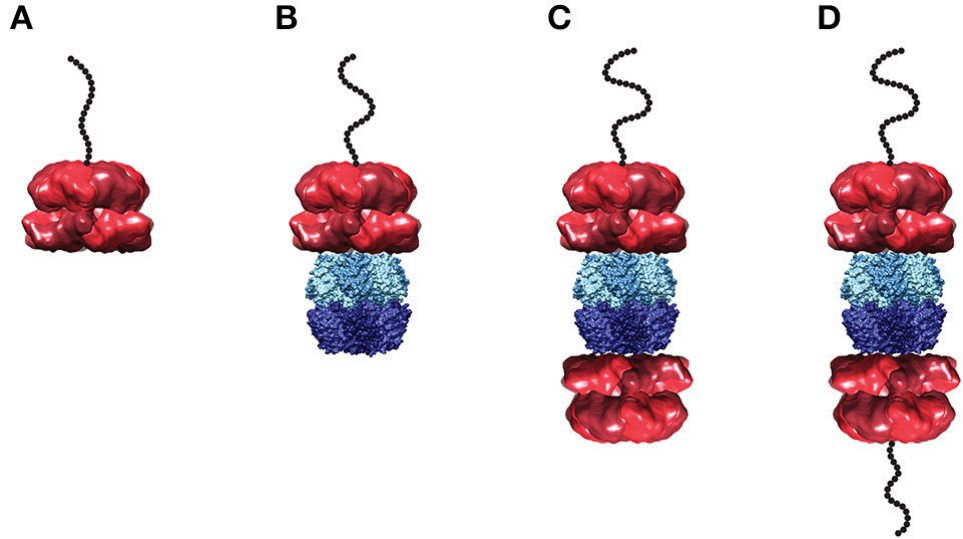
Structural models of ClpA and ClpAP complex in various states of peptide ligation. Models of **(A)** hexameric ClpA, **(B)** 1:1 ClpAP, and **(C)** 2:1 ClpAP bound by one peptide or **(D)** bound by two peptides. Structures are shown as side views in complex with a cartoon of an unstructured polypeptide substrate (black). The single particle reconstruction of *E. coli* ClpA hexamer (EMD-1673) (Effantin et al., 2010) is shown with monomers colored in alternating red shades. In the ClpAP complexes, a molecular surface from the crystal structure of *E. coli* ClpP tetradecamer (PDB-2FZS) (Szyk and Maurizi, 2006) is shown with protomers in the each heptameric ring colored in alternating shades of either light blue (top) or dark blue (bottom). The models shown here are not energy minimized. Images were prepared using UCSF Chimera (Computer Graphics Laboratory, University of California, San Francisco).

The single turnover stopped-flow method described above was employed to examine polypeptide translocation catalyzed by ClpAP. However, upon building a complex of polypeptide bound by ClpAP, a number of questions emerge. Hexameric ClpA can bind to either apical surface of ClpP forming a 1:1 complex, or to both apical surfaces of ClpP forming a 2:1 complex (see Figure 5). Should the experimental design conditions examine 1:1 or 2:1 hexameric ClpA to tetradecameric ClpP? Similarly, if the 2:1 complex is examined, should both sides of the enzyme be bound with peptide?

Based on activity measurements, Maurizi and coworkers reported an affinity for ClpA hexamer binding to ClpP tetradecamer to be ~4 nM (Maurizi et al., 1998). However, the fact that ClpA resides in a distribution of oligomers was not taken into account. ClpA resides in a distribution of monomers, dimers, and tetramers in the absence of nucleotide (Veronese et al., 2009; Veronese and Lucius, 2010). However, even at concentration of nucleotide above 1 mM there remains a distribution of oligomeric states (Veronese et al., 2011; Li and Lucius, 2013). Thus, it cannot be assumed that all of the ClpA present in solution is in the hexameric state.

For a macromolecule with two binding sites, one can be certain to ligate only one of the binding sites if the two-site macromolecule is maintained in large excess over the ligand. Thus, whether 1:1, 2:1 or a mixture of the ClpAP complexes are present in solution, by maintaining the complex in excess over the polypeptide only one peptide can be bound to any given ClpAP complex in the ensemble.

To build a peptide pre-bound complex, 86 nM tetradecameric ClpP and 1 μM monomer of ClpA were used in the presence of 150 μM ATPγS. Note that, unlike ClpA, ClpP forms stable tetradecamers (Maurizi et al., 1998) (E. Duran unpublished data). However, the question is; how much hexameric ClpA is present at 1 μM monomer? To address this question, sedimentation velocity experiments measured the concentration of hexameric ClpA in the presence of 150 μM ATPγS at 1 μM total ClpA monomer concentration. Under these conditions, the hexameric concentration was determined to be 130 nM. It is important to note that if the 1 μM total monomer concentration is divided by six, i.e., assume only hexamers are in solution, then one would predict 170 nM hexamers, an over estimate by 30% of the hexameric ClpA population. Under these conditions, a mixture of 1:1 and 2:1 complexes is predicted. With that in mind, binding the complex to 20 nM peptide maintains ClpAP (whether 1:1 or 2:1 complex) in large excess over the peptide. Keeping the ClpAP complex in excess over the peptide concentration ensures that peptide is only bound to one ClpA hexamer in a given ClpAP molecule. That is to say, it would be thermodynamically unfavorable to have a doubly peptide ligated 2:1 ClpAP complex.

Subjecting ClpAP to the same analysis as performed on ClpA alone revealed that, indeed, ClpAP does translocate with a faster overall rate of ~35 AA s^−1^ (Miller et al., 2013). This is ~1.5 times faster than the ~20 AA s^−1^ observed for ClpA alone. The overall rate is the product of the step size and the elementary rate constant governing that step. One of the strengths of the transient state kinetic approaches used is that it is sensitive to these two additional parameters. Interestingly, the kinetic step size for ClpAP was observed to be ~5 AA step^−1^ in stark contrast to the ~14 AA step^−1^ measured for ClpA alone (Rajendar and Lucius, 2010). Further, the rate constant governing translocation was found to be ~7 s^−1^, which is ~5-fold faster than the ~1.4 s^−1^ measured for ClpA (Miller et al., 2013).

As stated above, the kinetic step-size does not necessarily represent physical movement. However, a recent single-molecule examination of ClpAP translocation reports steps of ~1 nm (Olivares et al., 2014), which was reported to be consistent with the 5 AA step^−1^ reported from the single turnover experiments described above (Miller et al., 2013). A single molecule experiment that would be sensitive to mechanical movement has not been performed on ClpA alone. Such an experiment would either confirm or refute the measured ~14 AA step^−1^. Additional testing is necessary to determine whether or not this kinetic step-size represents mechanical movement.

All in all, it is clear that ClpP exerts an allosteric influence on ClpA catalyzed polypeptide translocation. Thus, ClpA and ClpAP should be considered to be two different enzymes that translocate with two different mechanisms. Moreover, questions remain regarding the activities of the 2:1 and 1:1 complexes.

The Walker A and Walker B motifs that form the ATP binding pocket are separated by a loop that extends into the axial channel of ClpA (Guo et al., 2002b). It has been proposed that the loop cycles up and down as the ATP binding site cycles through bound ATP to bound ADP + P_i_ and then release of ADP and P_i_. This up and down motion is thought to drive translocation. Hinnerwisch and coworkers showed through crosslinking studies that polypeptide substrate crosslinked with the NBD2 loop in the central channel of ClpA (Hinnerwisch et al., 2005). From these observations, Hinnerwisch and coworkers proposed that the NBD2 loop was responsible for mechanical pulling on the substrate polypeptide being translocated. They proposed a cycle of translocation to consist of ATP binding at NBD2 with the NBD2 loop in the up conformation, followed by ATP hydrolysis that drives movement of the NBD2 loop to the down conformation and concurrent movement of the polypeptide substrate that is bound to the NBD2 loop. Consistently, synchrotron footprinting data showed that the NBD2 loop proceeds through a nucleotide-dependent conformational change (Bohon et al., 2008).

From examination of the ATP concentration dependence of the kinetic step-size and rate constant for ClpAP, the observed step immediately follows ATP binding (Miller et al., 2013). Coupling this observation with the Hinnerwisch model, the step detected in the single-turnover experiments could be either ATP hydrolysis or a conformational change; a conformational change that may represent movement of the NBD2 loop. Since a single repeating step was detected in each cycle of translocation, loop movement may represent movement by ~5 amino acids.

If the measured kinetic step-size for ClpAP truly represents mechanical movement by ~5 amino acids then why does ClpA alone exhibit a different kinetic step-size of ~14 AA step^−1^? A potential answer to this question lies in the dependence of the overall translocation rate on [ATP] for ClpA and ClpAP. The translocation rate constant for ClpA alone exhibited a sigmoidal dependence on ATP. The isotherm could not be described by a simple rectangular hyperbola. Rather, it required analysis using a Hill model with a hill coefficient of ~2.5. In contrast, the translocation rate constant for ClpAP did not exhibit a sigmoidal dependence. Since ClpA contains two ATP binding sites per monomer and the single-turnover kinetic time courses are sensitive only to bound hexamer, the observation of a sigmoidal dependence suggests that there is cooperativity between multiple ATP binding sites that are involved in polypeptide translocation. On the other hand, since ClpAP did not exhibit any cooperativity, this indicates that the presence of ClpP relieves the cooperative interactions.

**Figure 6:**
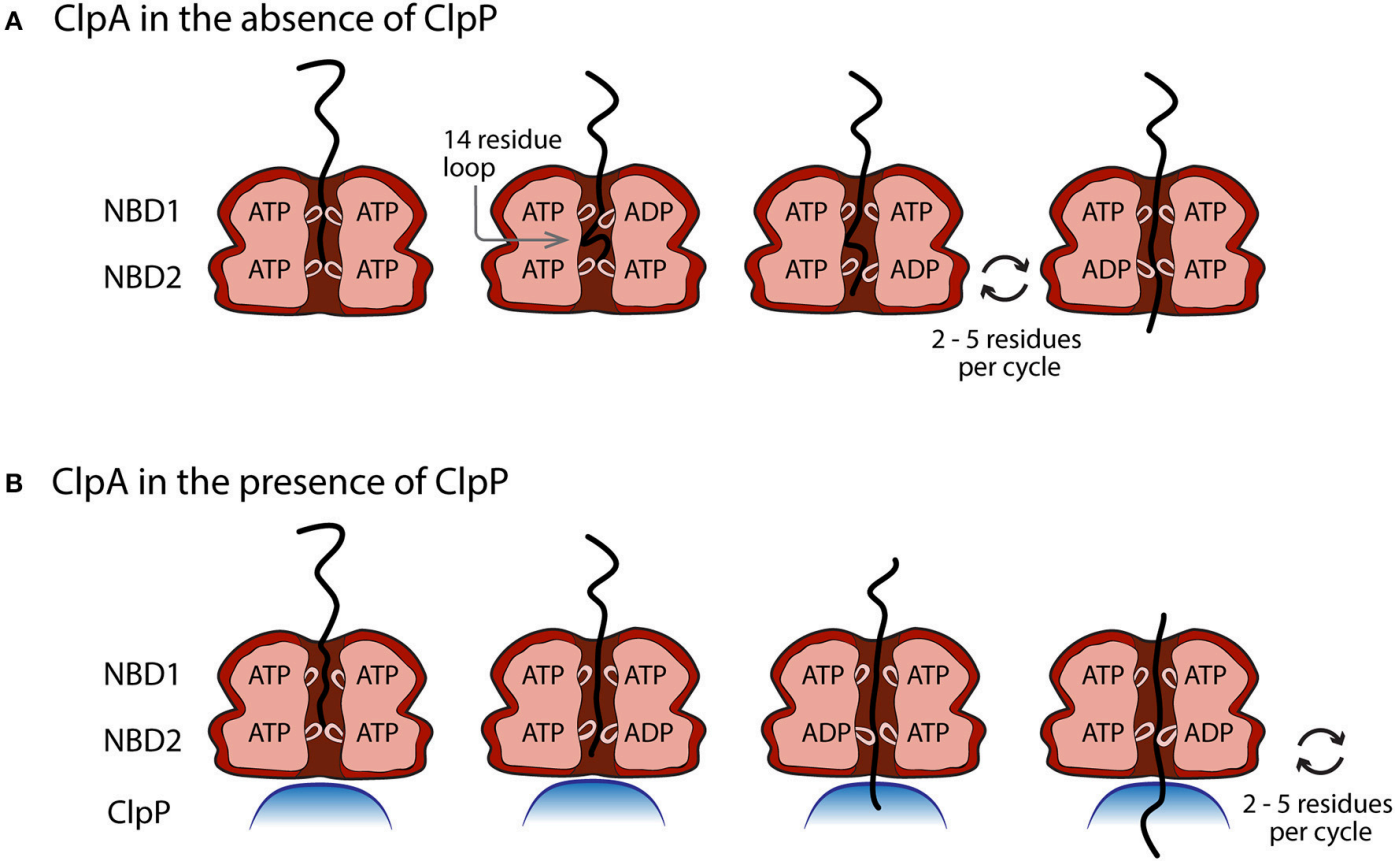
Proposed model of the movement of pore loops in NBD1 and NBD2 of ClpA in polypeptide translocation. **(A)** In the absence of ClpP, conformational changes in the pore loops of both NBD1 and NBD2 contribute to the translocation of polypeptide substrate through the ClpA axial channel. ATP binding and hydrolysis at NBD1 results in a pore loop conformational change that moves the incoming polypeptide substrate ~14 amino acids down the axial channel toward NBD2. This results in the formation of a polypeptide substrate loop in the axial space between NBD1 and NBD2. This loop is moved through the axial channel by multiple rounds of ATP hydrolysis cycles at NBD2 that lead to the translocation of 2–5 amino acids per cycle by the NBD2 pore loops. **(B)** In the presence of ClpP, polypeptide translocation is driven by NBD2 ATP hydrolysis induced conformational changes. Cycles of ATP binding and hydrolysis at NBD1, do not result in conformational changes that limit the observed ClpAP catalyzed translocation rate.

With these observations in mind, Figure 6 illustrates a working model for both ClpA and ClpAP polypeptide translocation, incorporating known structural information and various biochemical/biophysical studies. Figure 6A illustrates ClpA, in the absence of ClpP, with both the NBD1 and NBD2 loops in the up conformation and ATP bound to both domains. The polypeptide substrate is shown in black and is making contact with both the NBD1 and NBD2 loops. Crosslinking studies have shown that contacts between polypeptide substrate and ClpA were only observed with the NBD2 loop, but various single site mutations throughout the NBD1 loop abolished translocation activity (Hinnerwisch et al., 2005). Moreover, recent work indicates that both ATPase sites are involved in translocation catalyzed by ClpA in the absence of ClpP (Rajendar and Lucius, 2010). These two observations implicate the NBD1 loop in translocation. The next step would be for NBD1 to hydrolyze ATP and cause the NBD1 loop to move down and translocate (push) the substrate by up to 14 amino acids creating a polypeptide loop inside the axial channel of ClpA. The loop in the substrate can be accommodated in ClpA since it has been shown that ClpA forms a cavity between the NBD1 and NBD2 loops (Beuron et al., 1998; Guo et al., 2002a). NBD1 would contain ADP and P_*i*_ in the ATP binding site and therefore the NBD1 loop would have a reduced affinity for the polypeptide, which would allow for rebinding by another NBD1 loop loaded with ATP in a neighboring subunit in the hexamer (Farbman et al., 2007; Veronese et al., 2011). The NBD2 loop would cycle through multiple rounds of ATP hydrolysis coupled to translocation of the substrate by 2–5 amino acids per cycle with a rate constant of ~4 s^−1^. This will occur several times thereby shortening the loop inside the cavity of ClpA before NBD1 translocates another ~14 amino acids of the polypeptide into the cavity with a rate constant of 1.4 s^−1^.

Figure 6B illustrates the working model for how ClpA translocates when associated with ClpP. Since the ATP concentration dependence of the rate of ClpAP catalyzed polypeptide translocation suggests reduced cooperativity between ATP binding sites, it is hypothesized that NBD2 drives translocation in the ClpAP complex. Repeating cycles of ATP binding and hydrolysis could occur at NBD1, but they do not limit the observed translocation. Therefore, this model predicts repeating cycles of ATP binding and hydrolysis at NBD2 would lead to translocation of the substrate by distances of 2–5 aa step^−1^.

The working model predicts that in the absence of ClpP, NBD1 should hydrolyze ATP with a rate constant of (1.39 ± 0.06) s^−1^ and NBD2 should hydrolyze ATP with a rate constant of (7.9 ± 0.2) s^−1^ in the presence of polypeptide substrate. Kress et al. examined the steady state rate of ATP hydrolysis catalyzed by ClpA both in the presence and absence of ClpP (Kress et al., 2009). Further, they made two variants of ClpA that are deficient in ATP hydrolysis at either NBD1 or NBD2, which allow for the examination of ATP hydrolysis at each domain in the absence of hydrolysis at the other domain, and in the presence or absence of ClpP and SsrA substrate. Interestingly, in the absence of ClpP and the presence of GFP-SsrA, NBD1 hydrolyzes ATP with a rate constant of (0.8 ± 0.2) s^−1^, which is comparable to the rate constant determined for translocation of (1.39 ± 0.06) s^−1^ determined using the single-turnover stopped flow experiments. Similarly, in the presence of ClpP and GFP-SsrA, NBD2 hydrolyzes ATP with a rate constant of (6.3 ± 0.5) s^−1^, which is similar to the estimate of (7.9 ± 0.2) s^−1^ (Miller et al., 2013).

## Mechanism of translocation by ClpB/Hsp104

As stated above, ClpB/Hsp104 shares many structural characteristics with ClpA (see Figure 1) and therefore has been hypothesized to share a similar translocation mechanism. One important difference is the absence of an IGF/L loop in ClpB/Hsp104, necessary in ClpA for binding the protease ClpP. This structural difference intimates an important functional difference; ClpB/Hsp104 does not partner with any known protease (Woo et al., 1992).

A disaggregase such as ClpB/Hsp104 does not covalently modify its protein substrate. Disaggregation has been measured by monitoring changes in turbidity, solubility, and various staining techniques *in vitro*, thermotolerance development studies *in vivo*, and enzyme reactivation *in vivo* or *in vitro* (Parsell et al., 1991, 1994b; Glover and Lindquist, 1998; Goloubinoff et al., 1999; Zolkiewski, 1999; Mogk et al., 2003; Weibezahn et al., 2003; Schlee et al., 2004; Shorter and Lindquist, 2004; Schaupp et al., 2007; del Castillo et al., 2010; Sielaff and Tsai, 2010). These macroscopic observations, while informative, do not report on the molecular level events involved in the mechanism. How can the molecular events in the translocation or disaggregation mechanism be studied in the absence of a covalent modification to the protein substrate? Early investigations of the ClpB/Hsp104 disaggregation mechanism addressed this challenge by building upon the structural similarities between ClpB/Hsp104 and *E. coli* ClpA. As discussed above, ClpA processively translocates protein substrates through its axial channel and into the protease, ClpP. The similarities in sequence, tertiary structure, and quaternary structure lead the Bukau group to engineer the IGF/L loop onto the C terminal surface of ClpB and Hsp104. This loop allows a non-native interaction with ClpP, resulting in degradation of the substrate, a measurable covalent modification (Weibezahn et al., 2004; Tessarz et al., 2008). The rationale was that if they could “force” ClpB (Hsp104) to interact with ClpP and they observed proteolytic degradation, then this must mean that ClpB, like ClpA was translocating a substrate through the axial channel and into ClpP for proteolytic degradation.

In these studies, the Bukau group showed that the non-native BAP (ClpB-ClpA-P loop) -ClpP or HAP (Hsp104-ClpA-P loop) -ClpP complex was indeed able to degrade substrate proteins. This observation was interpreted as evidence that BAP and HAP, and therefore ClpB and Hsp104, processively translocate entire proteins through the axial channel and into ClpP, just as is done by the processive translocase ClpA (Weibezahn et al., 2004; Tessarz et al., 2008). Notably, additional studies of BAP-ClpP in which only portions of a substrate were unfolded lead the Bukau group to conclude, “partial threading of the unfolded substrate moiety through the central channel of ClpB is sufficient for efficient protein disaggregation in a physiologically relevant context” and that “partially threaded polypeptide chains are released from ClpB to be refolded” (Haslberger et al., 2008). Since these publications, however, many researchers in the field have often interpreted or summarized the Bukau results with less nuance, carrying forward only the “complete threading” model of polypeptide translocation.

The current prevailing hypothesis in the field is that the BAP-ClpP and HAP-ClpP findings, together with the structural similarities to ClpA, are evidence of complete threading or processive translocation by ClpB and Hsp104. The dominant mechanistic model is the translocation of an entire full-length protein pulled out of an aggregate through the axial channel of the disaggregating motor. The exclusive portrayal of this complete threading/processive translocation mechanism for these disaggregases has been schematized throughout the literature (Miot et al., 2011; Doyle et al., 2013). Other primary research has also been interpreted as consistent with the complete threading model based largely on the BAP/HAP–ClpP results (Schaupp et al., 2007; Nakazaki and Watanabe, 2014). It should be noted, however, that some researchers in the field do point out the possibility of both complete and partial threading mechanisms (Aguado et al., 2015).

Another important challenge to the findings using BAP and HAP with ClpP is that recent work has shown that BAP-ClpP degrades α-casein in both the absence and the presence of ATP (Li et al., 2015b). Thus, the degradation observed in this experimental design does not report strictly on the ATP-dependent translocation mechanism. Nakazaki and Watanabe's findings from their study of various mutations of *T*BAP-ClpP were interpreted as passive threading, independent of ATP hydrolysis (Nakazaki and Watanabe, 2014). However, these results could alternatively be understood to show that the *T*BAP-ClpP construct does not report exclusively on ATP-dependent translocation (threading) since they found “no correlation between ATPase activities and degradation rates” (Nakazaki and Watanabe, 2014).

A complementary approach to the BAP-ClpP degradation experiments, one in which there is no forced interaction with a protease, is needed. The stopped-flow fluorometer experimental design, described above (Figure 3), developed for the study of ClpA in the absence of ClpP is one such complementary approach (Rajendar and Lucius, 2010). Using this design, Li et al. demonstrated that ClpB is a non-processive translocase, taking only one or two kinetic steps before releasing the polypeptide substrate (Li et al., 2015b). This finding is at odds with the prevailing model of complete threading, by which one polypeptide chain is extracted from an aggregate. However, the Li et al. conclusion is in good agreement with previous results of observed partial threading (Haslberger et al., 2008). Additional studies are needed to expand this work into Hsp104.

Though Hsp104 and ClpB are both structurally and functionally similar, important differences have been observed. For example, both Hsp104 and ClpB can resolve disordered aggregates, however only Hsp104, not ClpB, can also resolve more structured amyloid aggregates (DeSantis et al., 2012). Hsp104 also has an additional function in prion curing not observed for ClpB (Shorter and Lindquist, 2004). What mechanistic differences give rise to these observations?

One possible contribution to the differences between the disaggregases is the differing roles of the two NBDs. The interplay between the NBDs within a hexamer is complex and cooperative. Still, some distinctions between NBD1 and NBD2 have been drawn. Notably, nucleotide binding at NBD1 is necessary for stabilization of ClpB hexamers (Kim K. I. et al., 2000; Watanabe et al., 2002; Mogk et al., 2003; del Castillo et al., 2010). This role of NBD1 in oligomerization is conserved between ClpB and ClpA. Surprisingly, in Hsp104, nucleotide binding in NBD2 is required for stabilization of hexamers (Parsell et al., 1994a; Schirmer et al., 1998).

In both ClpB and Hsp104, like in ClpA, the tyrosines in the pore loops of both NBDs are important for substrate processing (Schlieker et al., 2004; Weibezahn et al., 2004; Lee et al., 2007; Tessarz et al., 2008; Yokom et al., 2016; Gates et al., 2017). As the ATP hydrolysis cycle is carried out in either NBD, the pore loop is thought to move through space due to conformational changes induced by the nucleotide ligation state. The relatively large, planar surface of the tyrosine residue is thought to interact with the polypeptide substrate, pushing or pulling the polypeptide through the central channel. It's possible that differences in nucleotide binding/hydrolysis induced pore loop conformational changes account for the functional differences that exist between ClpB and Hsp104 catalyzed protein disaggregation. Experimental designs that report on the molecular level events involved in ClpB/Hsp104 polypeptide substrate processing, in particular those sensitive to the coordination between pore loop movement and nucleotide ligation state during disaggregation, will be key in testing this hypothesis.

### Effect of DnaK/Hsp70 on ClpB/Hsp104 mechanism

ClpB and Hsp104 were initially observed to disaggregate clients only in the presence of co-chaperones. These disaggregating motors are far more potent in collaboration with co-chaperones, although conditions have since been found in which ClpB and Hsp104 have innate disaggregation abilities. The co-chaperone system for *E. coli* ClpB is made up of DnaK, DnaJ, and the nucleotide exchange factor GrpE (termed the KJE system). Yeast Hsp104 collaborates with the co-chaperones Hsp70 (analogous to DnaK) and Hsp40 (analogous to DnaJ). Like ClpB/Hsp104, DnaK/Hsp70 is an ATPase and a disaggregase that can function independently of co-chaperones. The full systems, ClpB/KJE and Hsp104/70/40, have ATPase and disaggregase activity greater than the sum of the components' activities. There are three proposed possibilities that could explain this enhanced activity: (1) DnaK modifies the aggregate making a better binding site for ClpB, (2) DnaK accepts substrate from ClpB after the substrate has been completely translocated, or (3) the ClpB-DnaK complex has greatly amplified disaggregation activity relative to ClpB alone, possibly through a fundamentally different mechanism.

Early attempts to identify which component of the system acted upon an aggregate or client first resulted in divergent findings. The Liberek group identified DnaK as the first actor. They found that DnaK, with DnaJ and ATP, remodeled aggregates to facilitate ClpB-catalyzed disaggregation. Neither a transient tertiary complex with ClpB or additional roles for DnaK downstream of ClpB's action were ruled out (Zietkiewicz et al., 2004, 2006). On the other hand, early work from the Bukau group concluded that ClpB acted first. Specifically, ClpB was observed to expose a substrate's hydrophobic regions, which could then be recognized by the KJE system (Goloubinoff et al., 1999). The development of a ClpB trap mutant (double Walker B variant, able to bind but not hydrolyze ATP) also revealed that ClpB_trap_ outcompeted DnaK for binding to a model substrate and inhibited DnaK activity (Weibezahn et al., 2003).

Over time, the idea of a ClpB-DnaK (Hsp104-Hsp70) complex has come into favor. One compelling observation in support of this finding is that the activity of the co-chaperones is species specific. ClpB works with DnaK but not Hsp70. Hsp104 works with Hsp70 but not DnaK. This suggests a direct interaction between the chaperones. Furthermore, both the Wickner and Tsai groups engineered sets of chimeras in which domains from ClpB were replaced by the analogous domain from Hsp104 and vice versa. Both groups found that the M domain dictates which species formed a productive cochaperone partnership. For example, Hsp104 with the M domain from ClpB partnered effectively with the KJE system, not the Hsp70 system. This finding is consistent with the identification of the M domain as the binding site for DnaK (Miot et al., 2011; Rosenzweig et al., 2013; Doyle et al., 2015).

Binding affinities of 17 and 25 μM have been reported for *T. thermophilus* ClpB and DnaK (Schlee et al., 2004; Rosenzweig et al., 2013). For *E. coli* ClpB and DnaK, the K_*d*_ has been estimated in the range of 7–30 μM (Kedzierska et al., 2005). Notably, while the ClpB-DnaK complex has been observed by co-elution assays (Schlee et al., 2004; Barnett et al., 2005) and NMR (Rosenzweig et al., 2013), the ternary complex of ClpB-DnaK-client has not been observed. Furthermore, despite the K_*d*_ measurements and estimates in the range of ~20 μM, biochemical assays are often carried out with nanomolar to low micromolar concentrations of DnaK, conditions in which a significant population of the ClpB-DnaK complex is not expected (Weibezahn et al., 2004; Haslberger et al., 2008; DeSantis et al., 2012; Seyffer et al., 2012; Rosenzweig et al., 2013; Aguado et al., 2015; Doyle et al., 2015). Nevertheless, in these cases observations are attributed to the interplay between ClpB and DnaK. Though the existence of a DnaK-ClpB or Hsp70-Hsp104 complex has become widely accepted, the role of co-chaperones upstream and/or downstream of that complex remains under investigation. The convergence of evidence suggests that DnaK acts on the aggregate first, possibly targeting the client to ClpB, and then DnaK binds ClpB unleashing the disaggregating power of ClpB (Weibezahn et al., 2004; Sielaff and Tsai, 2010; Miot et al., 2011; Seyffer et al., 2012). DnaK may also have additional roles in the proper refolding of the client after release from ClpB.

## Conclusions

The reviewed studies reveal important considerations for design and implementation of the experiments needed to address outstanding questions about ClpA and ClpB/Hsp104 catalyzed protein translocation, degradation, and disaggregation mechanisms, respectively. One major aspect of assay design is the ability to predict the population of degradation/disaggregation active complex present under the chosen experimental conditions. As work on ClpB has revealed, these proteins persist in a distribution of oligomers even at high nucleotide concentrations (Figure 2). Therefore, dividing the monomeric protein concentration by six will yield overestimates for the hexameric population present and available to interact with partner proteins and substrates in solution. Instead, quantification of the active hexamer population in a given assay will require a thermodynamically rigorous characterization of the energetics governing nucleotide-linked self-assembly. Although this work has been done for ClpB, the mechanisms of ClpA and Hsp104 ligand linked self-assembly remain to be examined.

A related consideration in assay design is the effect of mutations on AAA+ motor self-assembly. Because the propensity of a protein to oligomerize is in part driven by its primary sequence, mutations of the sequence will have effects on its self-assembly. If unaccounted for, assay readout changes resulting from up- or downregulation of the hexamer population as a result of mutations, could be misinterpreted as up- or downregulation of “activity” in ATPase, reactivation, or other assays. Thus, when designing experiments for AAA+ motors and their corresponding variants, it is important to know whether the signal being monitored reports on events that could be controlled by changes in the assembly state. Interpretations of those results should be tempered by possible contributions from variability in the assembly state.

Single turnover translocation experiments have been designed to yield information about the molecular level events governing AAA+ motor activity without rigorous quantification of the self-assembly mechanism (Rajendar and Lucius, 2010; Li et al., 2015b). However, this was possible, in part, because only hexamers are bound to the polypeptide substrate. If smaller oligomers contributed to the translocation signal then measures would have to be taken to account for this. For example, as soon as ClpP is introduced to ClpA then one has to start asking how the distribution of 1:1 and 2:1 hexameric ClpA to tetradecameric ClpP influences the signal. Similar techniques are being adopted to investigate the molecular level events governing the mechanism of ClpB/Hsp104 catalyzed disaggregation in the absence and presence of partner co-chaperones. As work on ClpA and ClpAP revealed, ClpP induces a major change in the mechanism of ClpA catalyzed polypeptide translocation. It's reasonable, then, to expect cochaperones like DnaK/Hsp70 to similarly affect the disaggregation mechanism of ClpB/Hsp104. Implementation of these transient state kinetic techniques will prove powerful in the deconvolution of cochaperone contributions to the disaggregation activities of ClpB/Hsp104 and functional differences between ClpB and Hsp104.

By definition, motor proteins use an energy source to perform mechanical work. ClpA and ClpB/Hsp104 use the energy from ATP binding/hydrolysis to perform this mechanical work. For any translocase there is interest in how far the translocase moves on its lattice, how much energy is required to make this movement, and how much force is exerted. For ClpA we reported the first kinetic step-size for any AAA+ protein translocase to be ~14 amino acids per step (Rajendar and Lucius, 2010). Similarly, we showed that ClpAP translocated with a reduced kinetic step-size of ~5 amino acids per step (Miller et al., 2013). Consistently, a single molecule optical tweezer measurement reported a step-size of ~5 amino acids per step for ClpAP (Olivares et al., 2014). Similarly, single molecule optical tweezer experiments showed that ClpXP translocated in 5–8 amino acid steps (Aubin-Tam et al., 2011; Maillard et al., 2011). In many cases, single-molecule and single turnover kinetics experiments can get around the limitations on the interpretation imposed by macromolecular assembly. Thus, going forward, the combination of single-molecule and transient state kinetic experiments are going to be essential for addressing detailed mechanistic questions on AAA+ motors.

## Author contributions

ED, CW, and AL contributed equally to this work. ED and CW are co-first authors.

### Conflict of interest statement

The authors declare that the research was conducted in the absence of any commercial or financial relationships that could be construed as a potential conflict of interest.
